# Commentary: the first twelve years of the *Journal of chemoinformatics*

**DOI:** 10.1186/s13321-022-00617-4

**Published:** 2022-06-13

**Authors:** Peter Willett

**Affiliations:** Information School, University of Sheffield, Sheffield, S10 2TN UK

**Keywords:** Bibliometric profile, Citations, *Journal of Cheminformatics*, Knowledge export

## Abstract

This commentary provides an overview of the publications in, and the citations to, the first twelve volumes of the *Journal of Cheminformatics*, covering the period 2009–2020. The analysis is based on the 622 articles that have appeared in the journal during that time and that have been indexed in the Clarivate *Web of Science Core Collection* database. It is clear that the journal has established itself as one of the most important publications in the field of cheminformatics: it attracts citations not only from other journals in its specialist field but also from biological and chemical journals more widely, and moreover from journals that are far removed in focus from it but that are still able to benefit from the articles that it publishes.

Writing in 2009 in the very first paper published in the *Journal of Cheminformatics* (hereafter *JCheminf*), the Editor-in-Chief, David Wild, noted that cheminformatics was a discipline “that has a history longer than most applied computational disciplines; that has elegantly solved so many basic (and not so basic) problems; that has a reputation for intellectual rigour and good-naturedness; that has hundreds of scholarly articles published; and that has impacted fields as diverse as drug discovery, library science and database searching” [1]. While the discipline was clearly of increasing importance, the journal was established to provide a focus for a field that was then widely but thinly spread, involving a diverse group of researchers who were working in both academe and industry, who came from many different scientific backgrounds, and who were reporting their research in a wide range of academic publications [1, 2].

In this brief commentary, the methods of bibliometrics are used to study the extent to which the journal has been successful in providing a focus for the discipline. Bibliometrics—sometimes called informetrics or scientometrics, although the methods are increasingly not restricted just to the sciences—involves the quantitative analysis of data associated with the published literature. The data is most commonly numbers of publications, citations or downloads; and this is used to identify, e.g., the extent of authors’ contributions to a discipline, new measures that can quantify the impact of research, and the extent to which research in one field can influence research in another [3–6].

The present author and colleagues have previously published several bibliometric studies of the field of cheminformatics, e.g. [2, 7–10]. The present report extends those studies with an analysis of the articles in, and the citations to, the first twelve volumes of *JCheminf*, covering the period 2009–2020; there is also reference, where appropriate, to the most recent articles published in the journal 2021 [11]. The analysis is based on the articles that have appeared in the journal and that have been indexed in the Clarivate *Web of Science Core Collection* (hereafter WoS) database [12], which provides coverage since the very first issue of the journal. After the removal of editorial material, reviews and corrections etc., a search of WoS in early January 2022 identified a total of 622 articles that had been published in *JCheminf* by the end of 2020.

The frequencies of bibliometric data generally follow a power-law distribution and are hence often highly skewed, with a few items in a dataset occurring very often but with most occurring very infrequently [13]. This behaviour is exemplified by the 622 articles here: these were the work of 1907 different authors but the great majority (76.2% of them) were involved in just a single article. Conversely, eleven of the authors provided ten or more contributions, these comprising many important workers in the field (Andreas Bender, Evan Bolton, Stephen Bryant, Ola Engkvist, Sunghwan Kim, Peter Murray-Rust, Jean-Louis Reymond, Ola Spjuth, Christoph Steinbeck, Gerard van Westen, and Antony Williams). These authors have between them—after taking account of a few joint publications—contributed no less than 18.0% of the total number of *JCheminf* articles. An analogous, highly skewed distribution is obtained when considering the national affiliations of the authors’ institutions. There are 67 countries represented, with 17 of them providing just a single article, and with the distribution dominated first by the USA and then by the UK, with 182 and 119 articles respectively. There are only two further countries that have contributed to 50 or more articles in the journal: Germany with 90 and the People’s Republic of China with 57. The latter’s contributions have grown rapidly throughout the review period: there were none until 2012, but 10 in both 2019 and 2020 (and a further 14 in 2021).

There has been a fair degree of consistency in the subject matter of the articles. Figures 1(a) and (b) show the 50 most important title words for the periods 2009–2014 and 2015–2020, respectively (arranged using the EdWordle software [14], where the size of each word in a word-cloud reflects its frequency of occurrence within the titles after the removal of stop-words). While some minor differences between the two figures are evident, differences are much more obvious if one considers the word-cloud illustrating the titles for the 96 most-recent articles that appeared in 2021. This is illustrated in Fig. 1(c), where there are multiple words reflecting the current intense interest in the application of AI techniques to cheminformatics (and, of course, to science more generally). Indeed, no less than 54 of these 96 articles were returned in a search for (“artificial intelligence” OR “deep learning” OR “machine learning” OR “neural network”).

**Fig. 1:**
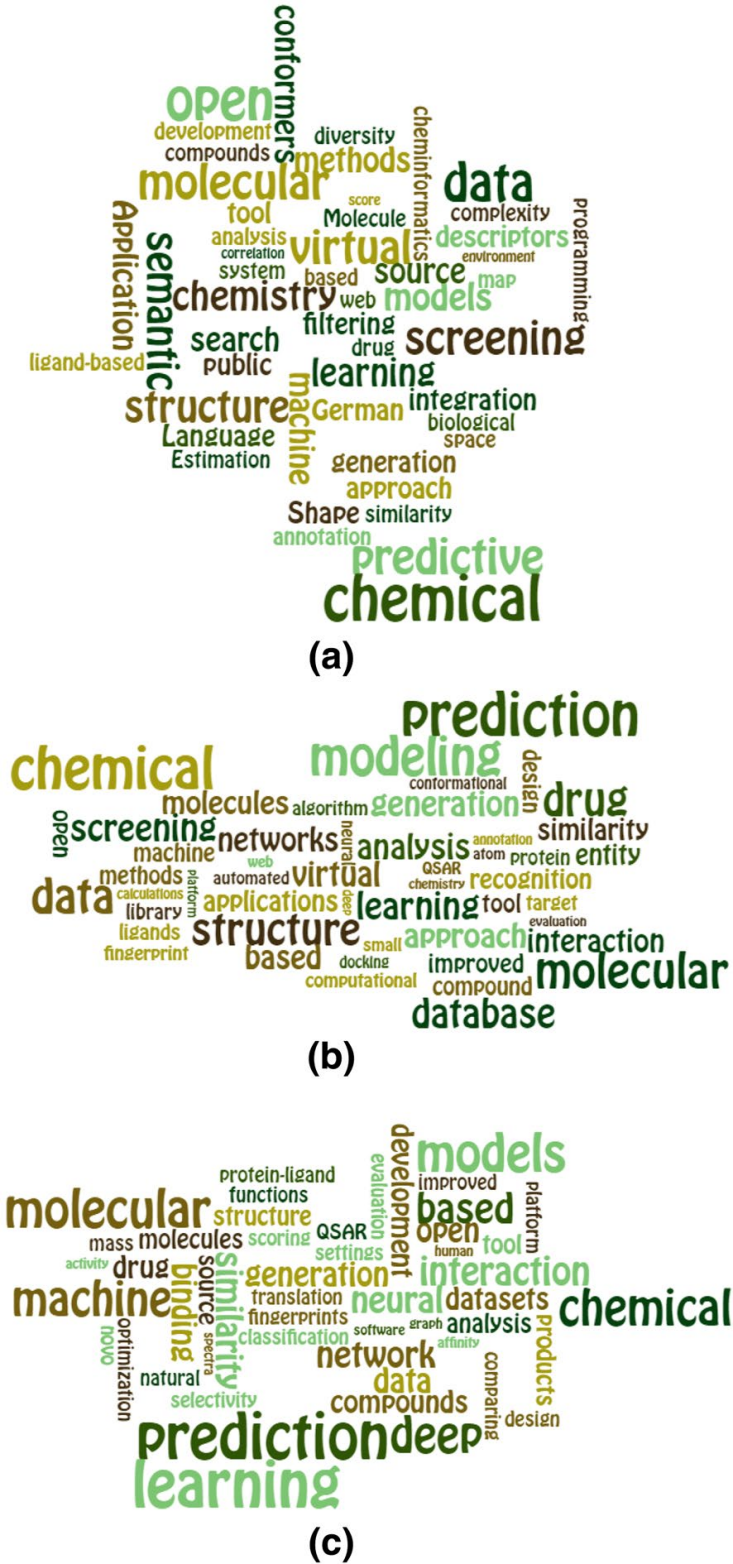
EdWordle plots for the 50 most prominent title words for (**a**) 2009–2014, (**b**) 2015–2020, and (**c**) 2021

The 622 articles had attracted a total of 17,889 citations up to the end of 2020 in WoS, a mean of 28.8 citations per article. As shown in Fig. 2, the number of citations has grown rapidly year on year as more and more articles become available for citation. Unsurprisingly there were just 5 in 2009, but 1346 in 2015 (the first year to yield more than a thousand citations) and then 4711 in 2020. The citations come from a total of 13,548 distinct citing articles, only 446 of which come from *JCheminf* itself, i.e., the total has not been boosted artificially by large numbers of journal self-citations [15].

**Fig. 2:**
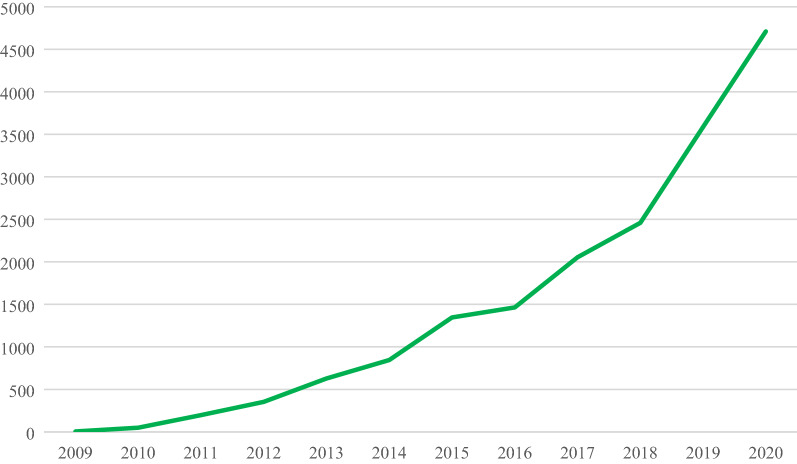
Citations to the journal from 2009 till 2020

As with the author and country distributions, that for the citation frequencies is extremely skewed, with the ten most cited articles listed in Table 1 providing 42.3% of the total citations to the journal. There is an obvious bias to older articles in a listing such as this, since they have had a greater period in which they can attract citations; even so, the dominance of the top two articles—which were published a decade ago—is striking since they account for no less than 28.7% of the total citations. Both of these papers describe software systems, and such papers have always attracted large numbers of citations as a piece of software becomes well established and increasingly used throughout the community [2]. Download statistics provide an additional measure of importance, with the articles quoting more than 270,000 downloads of Avogadro and more than 160,000 downloads of Open Babel. Table 1 contains two more software articles, describing the MetFrag and Confab systems, and the next ten highest-cited articles include a further four such descriptions (for MOLE 2.0, CDK, JSME and tmChem). Similar comments apply to database articles, such as the third-ranked one in Table 1 describing TCSMP. Given the increasing use of standard tools and open data, this behaviour will surely continue to be the case, especially as the open-access nature of the journal means that it is available to the entire research community, something that may enhance the citation counts still further [16].

**Table 1:** The ten articles attracting the largest numbers of citations up to the end of 2020

Hanwell MD et al. (2012) Avogadro: an advanced semantic chemical editor, visualization, and analysis platform	J Cheminform 4:17 (2697 citations)
O’Boyle NM et al. (2011) Open Babel: an open chemical toolbox	J Cheminform 3:33 (2445 citations)
Ru J et al. (2014) TCMSP: a database of systems pharmacology for drug discovery from herbal medicines	J Cheminform 6:13 (640 citations)
Bikadi Z, Hazai E.(2009) Application of the PM6 semi-empirical method to modeling proteins enhances docking accuracy of AutoDock	J Cheminform 1:15 (327 citations)
DeHaven CD et al. (2009) Organization of GC/MS and LC/MS metabolomics data into chemical libraries	J Cheminform 2:9 (319 citations)
Ruttkies C et al. (2016) MetFrag relaunched: incorporating strategies beyond in silico fragmentation	J Cheminform 8:3 (276 citations)
Bajusz D et al. (2015) Why is Tanimoto index an appropriate choice for fingerprint-based similarity calculations?	J Cheminform 7:20 (255 citations)
Krstajic D et al. (2014) Cross-validation pitfalls when selecting and assessing regression and classification models	J Cheminform 6:10 (212 citations)
Ertl P, Schuffenhauer A. (2009) Estimation of synthetic accessibility score of drug-like molecules based on molecular complexity and fragment contributions	J Cheminform 1:8 (200 citations)
O'Boyle NM et al. (2011) Confab—Systematic generation of diverse low-energy conformers	J Cheminform 3:8 (188 citations)

The ten journals providing the largest numbers of citations to *JCheminf* are *Journal of Chemical Information and Modeling* (647 citations), *JCheminf* itself (the 446 mentioned above), *Scientific Reports* (263), *Molecules* (217), *PLOS ONE* (198), *Bioinformatics* (163), *Journal of Biomolecular Structure and Dynamics* (161), *Physical Chemistry Chemical Physics* (156), *Molecular Informatics* (155), and *International Journal of Molecular Sciences* (136). It is hardly surprising that the *Journal of Chemical Information and Modeling* provides the largest number of citations to *JCheminf* since this has for long been the “core” journal for the field [2, 10]: it started life as the *Journal of Chemical Documentation* as far back as 1960, years before the arrival of any of the other journals.

A recent review of the literature of cheminformatics [10] suggested that the *Journal of Chemical Information and Modeling* and *Molecular Informatics* are the only other journals apart from *JCheminf* that have a noticeably strong focus on this specific topic, and the many citations from these journals are hence to be expected. The fact that large numbers are also obtained for the other journals listed here demonstrates that *JCheminf* is attracting interest from journals that are beyond, albeit clearly related to, its specialist field. Of those above, the only possible outlier given the general nature of its contents is *Physical Chemistry Chemical Physics*, but even here the top two articles in Table 1 are cited very frequently (with 89 and 24 citations respectively to the Avogadro and Open Babel articles).

The importance of a journal is often quantified by its Journal Impact Factor (or JIF), which is calculated by Clarivate for WoS journals. Given the number of citations received in a particular year to articles published in a particular journal during the two preceding years, then the JIF is the ratio of that number to the total number of articles published in the journal during those two preceding years. The 2020 JIF values for the three cheminformatics journals (*Journal of Chemical Information and Modeling*, *Molecular Informatics* and *JCheminf*) are 4.956, 3.353 and 5.514 respectively. It must be emphasized that there are many criticisms of the JIF when used as a quality criterion [17, 18] but the values here do suggest that *JCheminf* is at least comparable in its perceived standing to its two main competitor journals.

In all, the journal has received citations from 2829 different publications (mainly journals), some of which would appear to describe work in fields that are far removed not just from cheminformatics but from chemistry and biology more generally. This is an example of what has been called a knowledge export [19], i.e., the transfer of knowledge from one academic field to another. The extent of this behaviour can be quantified by using the WoS subject categories: here, every journal is allocated to one or more of 254 different categories, and it is hence possible to explore the extent of knowledge exports from *JCheminf* by considering the subject categories of the articles that cite it.

The *JCheminf* articles have been cited by journals belonging to no less than 200 different categories, some of which seem, on first sight at least, to have nothing to do with cheminformatics. For example, the 212 citations to the article by Krstajic [20] on the use of cross-validation for assessing classification and regression models include ones from journals as diverse as *Child Abuse & Neglect* (in the Social Work category) [21]), *Maritime Policy & Management* (in Transportation) [22]) and *Resuscitation* (in Emergency Medicine) [23]). There are many other such non-obvious citations: for example, an article in *Global Change Biology* (in Biodiversity Conservation) [24] made use of the OpenBabel toolbox article [25]; and one in *Computers, Environment and Urban Systems* (in Regional and Urban Planning) [26] drew on the work of Skuta et al. [27] on the visualization of dendrograms. It must be emphasized that the great majority of citations are from journals with which *JCheminf* might be expected to share at least some commonality of interest (as discussed previously); even so, the presence of citations such as the examples above serve to demonstrate the increasing breadth of the journal’s influence.

In conclusion, it would appear that the *Journal of Cheminformatics* has established itself as one of the most important publications in the field since it first appeared in 2009. It attracts attention (in the form of citations) not only from other journals in its specialist field, but also from biological and chemical journals more widely, and from journals that are far removed in focus from it but that are still able to benefit from the articles that it publishes.

## Data Availability

Not applicable.

## Abbreviations

*JCheminf*: *Journal of* *C**heminformatics*
JIF: Journal Impact Factor
WoS: Web of science

## References

[1] Wild DJ (2009). Grand challenges for cheminformatics. J Cheminform.

[2] Willett P (2008). A bibliometric analysis of the literature of chemoinformatics. Aslib Proc.

[3] Bar-Ilan J (2008). Informetrics at the start of the 21st century—a review. J Informetr.

[4] Mingers J, Leydesdorff L (2015). A review of theory and practice in scientometrics. Eur J Operat Res.

[5] Sugimoto CR, Larivière V (2018). Research: what everyone needs to know.

[6] Aksnes DW, Langfeldt L, Wouters P (2019). Citations, citation indicators, and research quality: an overview of basic concepts and theories. SAGE Open.

[7] Al Jishi R, Willett P (2010). The journal of chemical documentation and the journal of chemical information and computer sciences: publication and citation statistics. J Chem Inf Model.

[8] Wong R, Willett P, Allen FH (2010). The scientific impact of the cambridge structural database: a citation-based study. J Appl Crystal.

[9] Restrepo G, Willett P (2017). A bibliometric profile of MATCH communications in mathematical and in computer chemistry. MATCH Comm Math Comp Chem.

[10] Willett P (2020). The literature of chemoinformatics, 1978–2018. Int J Mol Sci.

[11] Brief references are also made to the 2021 publications in *JChemInf*. The data for this study was collected early in January 2022, at which point delays in journal publication and subsequent database processing mean that it is likely that the citation counts for 2021 are incomplete. The citation data hence covers only the period 2009–2020.

[12] Birkle C, Pendlebury DA, Schnell J (2020). Web of Science as a data source for research on scientific and scholarly activity. Quant Sci Stud.

[13] Newman MEJ (2005). Power laws, Pareto distributions and Zipf’s law. Contemp Phys.

[14] EdWordle. http://www.edwordle.nt. Accessed 27 Jan 2022.

[15] Heneberg P (2016). From excessive journal self-cites to citation stacking: analysis of journal self-citation kinetics in search for journals, which boost their scientometric indicators. PLoS ONE.

[16] Langham-Putrow A, Bakker C, Riegelman A (2021). Is the open access citation advantage real? A systematic review of the citation of open access and subscription-based articles. PLoS ONE.

[17] Seglen PO (1997). Why the impact factor of journals should not be used for evaluating research. BMJ.

[18] Paulus FM, Cruz N, Krach S (2018). The impact factor fallacy. Front Psychol.

[19] Yan E, Ding Y, Cronin B (2013). A bird’s-eye view of scientific trading: dependency relations among fields of science. J Informetr.

[20] Krstajic D, Buturovic L, Ljubomir J (2014). Cross-validation pitfalls when selecting and assessing regression and classification models. J Cheminform.

[21] Carmel T, Widon CS (2020). Development and validation of a retrospective self-report measure of childhood neglect. Child Abuse Neglect.

[22] Le LT, Lee G, Park KS (2020). Neural network-based fuel consumption estimation for container ships in Korea. Maritime Policy Manag.

[23] Rad AB, Engan K, Katsaggelos AK (2016). Automatic cardiac rhythm interpretation during resuscitation. Resuscitation.

[24] Roggatz CC, Lorch M, Hardege JD (2016). Ocean acidification affects marine chemical communication by changing structure and function of peptide signaling molecules. Global Change Biol.

[25] O'Boyle NM, Banck M, James CA (2011). Open babel: an open chemical toolbox. J Cheminform.

[26] Storme T, Derudder B, Dorry S (2019). Introducing cluster heatmaps to explore city/firm interactions in world cities. Comput Environ Urban Syst.

[27] Skuta C, Bartunek P, Svozil D (2014). InCHlib—interactive cluster heatmap for web applications. J Cheminform.

